# Toxicity of Three Insecticides to *Lysiphlebus fabarum*, a Parasitoid of the Black Bean Aphid, *Aphis fabae*

**DOI:** 10.1673/031.011.10401

**Published:** 2011-08-22

**Authors:** Qodratollah Sabahi, Arash Rasekh, J.P. Michaud

**Affiliations:** ^1^ Department of Plant Protection, College of Agriculture, University of Tehran, Daneshkade St, Karaj, Iran; ^2^ Department of Plant Protection, College of Agriculture, Shahid Chamran University of Ahvaz, Ahvaz, Iran; ^3^ Kansas State University, Agricultural Research Center — Hays, 1232 240th Ave, Hays, KS 67601, USA

**Keywords:** abamectin, imidacloprid, persistence, pymetrozine, *Vicia faba*

## Abstract

The toxicity of three insecticides to *Lysiphlebus fabarum* (Marshall) (Hymenoptera: Braconidae: Aphidiinae), a parasitoid of *Aphis fabae* Scopoli (Hemiptera: Aphididae), was investigated using IOBC/wprs protocols. Abamectin 1.8 EC, imidacloprid 350 SC, and pymetrozine 25 WP were tested under laboratory conditions at recommended field rates. Immature stages of the parasitoid were exposed to materials by briefly dipping mummified aphids into insecticide solutions/suspensions or water (controls). Abamectin, imidacloprid, and pymetrozine caused 44.8, 58.5, and 14.5% mortality of mummies, respectively. Insecticides were also applied to broad bean foliage until run-off using a hand sprayer and the contact toxicity of residues was investigated after 1, 5, 16 and 30 day periods of outdoor weathering by caging adult wasps on treated plants for 24 h. One day-old residues of abamectin, imidacloprid, and pymetrozine produced 52.5, 90.0 and 57.0% mortality, respectively, and 5 day-old residues produced 28.1, 77.0 and 18.6% mortality. Sixteen day-old residues produced 8.8, 22.4 and 13.6% mortality, whereas 30 day-old residues produced 0.0, 3.2 and 1.1% mortality, respectively. On the basis of these results, abamectin and pymetrozine were classified as short-lived compounds (Class A) and imidacloprid as a slightly persistent compound (Class B).

## Introduction

Aphids are an important group of plant pests with high reproductive potential. They inflict both direct and indirect damage to plants by extracting photosynthate and transmitting viruses. The black been aphid, *Aphis fabae* Scopoli (Hemiptera: Aphididae) is one of the most polyphagous aphid species, exploiting more than 200 leguminous plants and infesting all plant parts (Barnea et al. 2005). In broad bean, *Vicia faba* L. (Fabales: Fabaceae), growth may be diminished and flowers may abort in response to *A. fabae* saliva (Nuessly et al. 2004). Apterous virginoparae of *A. fabae* are able to overwinter in regions with a mild climate, allowing the species to survive without a sexual phase, or holocycle (Aghajanzadeh et al. 1997).

*Lysiphlebus fabarum* (Marshall) (Hymenoptera: Braconidae: Aphidiinae) is a specialized parasitoid of *A. fabae* on both crops and weeds and is ubiquitous in many agroecosystems (Starý 1986; Hildebrand et al. 1997; Völkl and Stechmann 1998; Raymond et al. 2000, Nuessly et al. 2004), including those in northern Iran (Matin et al. 2005). Asexual populations of this parasitoid are known in central Europe (Nemec and Starý 1985) and were recently reported in Iran (Rasekh et al. 2009). Members of the subfamily Aphidiinae complete preimaginal stages inside the body of the aphid (Marulle, 1987; Völkl & Stechmann, 1998; Carver 1989; Starý 1999).

Parasitoids have often been shown to be more sensitive to synthetic insecticides than their hosts. In order to integrate the use of biological control with pesticide applications, synthetic pesticides should be selected for minimal impact on biological control agents. Determination of the compatibility of pesticides with biological control requires information on their direct and indirect toxicity to beneficial species, the pest's economic threshold, and the timing of applications (Stark et al. 2007). Many of the conventional insecticides in current use are broad-spectrum neurotoxins that affect both target and non-target species and, as a result, may disrupt biological control processes (Talebi et al. 2008).

Insecticides commonly used for control of aphids on legume crops in Iran include abamectin, imidacloprid and pymetrozine. Abamectin is a natural fermentation product of the soil bacterium *Streptomyces avermitilis* and is used to control insect and mite pests of fruit, vegetable and field crops (Lankas and Gordon 1989). Abamectin acts on insects by interfering with neural and neuromuscular transmission (Hayes and Laws 1990). Imidacloprid is a nitroguanidine insecticide that is registered in a variety of formulations (Mullins 1993) and shows excellent activity on a variety of insect pests, including aphids, leafhoppers, plant hoppers, thrips and whiteflies (Elbert et al. 1990, Woodford and Mann 1992). Imidacloprid overstimulates nerve conduction in insects by mimicking the action of the neurotransmitter, acetylcholine (Mullins 1993). Whereas many biological control agents are susceptible to these older insecticides, some newly developed compounds claim to be less toxic to natural enemies. Pymetrozine belongs to a novel pesticide chemistry known as pyridine azomethines. It is highly selective because of a unique mode of action that acts specifically on the salivary pump of sucking insects causing rapid cessation of feeding. It is slow acting, has some translaminar activity, and is generally toxic to aphids and, to a lesser extent, whiteflies (Harrewijn and Kayser 1997).

Standardized methods involving both laboratory and field tests have been developed to test the safety of pesticides to beneficial organisms in accordance with IOBC guidelines (Hassan 1998). The objective of this research was to determine the susceptibility of *L. fabarum* to abamectin, imidacloprid, and pymetrozine, the insecticides commonly applied against *A. fabae* in greenhouses.

## Materials and Methods

### Insect colonies

A thelytokous colony of *L. fabarum* was established from mummies collected from black bean aphids infesting broad bean in a field in Zanjan Province, Iran, in June 2007. A stock colony of *A. fabae* was maintained on potted broad bean, *V. faba* var. Sarakhsi, grown in pots filled with fertilized sawdust in growth chambers set at 20 ± 1° C, 65–75% RH, and a 16:8 L:D photoperiod. The parasitoid was reared on *A. fabae* feeding on broad bean under the same conditions.

Cohorts of wasps were produced by exposing second instar *A. fabae* to female wasps in a 5:1 ratio in ventilated plastic cylinders (8.0 cm diameter × 20.0 cm length) for a period of six hours and then transferring the aphids to potted bean plants in a growth chamber until mummies formed about nine days later. Mummies were carefully removed from plants and put in plastic Petri dishes (9.0 cm diameter) until emergence, whereupon each adult female was released into a ventilated plastic cylinder (3.5 cm diameter × 7.0 cm length) provisioned with diluted honey (as droplets on a strip of wax paper) and water (on a cotton roll). The water was replenished daily and the diluted honey every two days. Unless otherwise noted, all females had continuous access to food prior to testing and were used in experiments when they were 72 ± 4 h old without prior exposure to aphids. All experiments were conducted in a walk-in growth chamber.

### Insecticides

The insecticides used in this study were commercial preparations of imidacloprid (Bayer Agricultural Products, www.bayercropscience.com) (350 SC, 40 mg ai per liter), pymetrozine (Syngenta AG, www.syngenta.com) (25 WP, 150 mg ai per liter), and abamectin (Gyah Corporation, Iran) (1.8 EC 20 mg ai per liter). The insecticides were each dissolved in tap water to produce concentrations equivalent to field rates, assuming an application volume of 400 liters per hectare.

### Preimaginal exposure

Broad bean leaves containing around 100 recently mummified aphids each were dipped in each insecticide solution, four leaves per treatment, with tap water used as a control. Treated mummies were then transferred to plastic Petri dishes in a fume hood and allowed to dry for one hour, before being transferred to a rearing room under conditions of 23 ± 1° C and 70 ± 5% RH until emergence of parasitoids approximately seven days later.

### Adult exposure

The direct toxicity and persistence of insecticides to three day-old adult wasps was evaluated under semi-field conditions. The insecticides were applied to potted broad bean foliage at the same rates as above using a hand sprayer until run-off and the plants were left exposed to the natural elements outdoors during the aging period. Pots were placed in plexiglass drum-cells (9.0 cm diameter × 5.0 cm height) for exposure of adult parasitoids in a rearing room under the conditions previously described. Ventilation was provided via eight holes (1.0 cm diameter) located around the side of the cage, each covered with fine gauze. Square glass plates (10 cm × 10 cm) were placed above and beneath each cage and 10 female wasps were introduced into each cage. Parasitoids were provided with diluted honey on a dental wick during tests and mortality was evaluated after 24 hours. Three replicates of 12 cages each were performed for each treatment. The cages were maintained in an environmental chamber under the standard rearing conditions.

### Statistical analysis

The experiment was arranged in an RCBD design consisting of three replicates per treatment. Treatment mortalities were adjusted for control mortality using Abbott's correction (Abbott 1925). Treatment effects were analyzed using ANOVA and means were separated using Fisher's LSD test (SAS 2001).

## Results

### Preimaginal exposure

**Figure 1.**
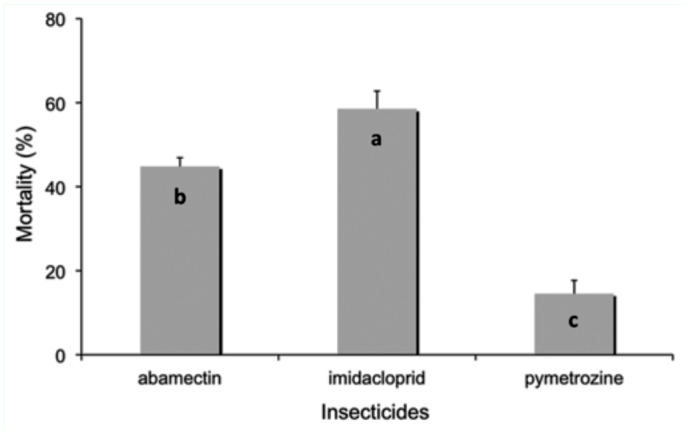
Mortality of *Lysiphlebus fabarum* females after topical exposure to three pesticides in the mummy stage. Means bearing different letters are significantly different (LSD, α = 0.05). High quality figures are available online.

*Lysiphlebus fabarum* mortality after exposure to imidacloprid, pymetrozine and abamectin in the mummy stage varied significantly among treatments (*F* = 164.18; df = 2; *p* < 0.001; Figure 1). According to IOBC standards, pymetrozine was classified as harmless, whereas imidacloprid and abamectin were classified as slightly harmful.

### Adult exposure

The mortalities of three day-old *L. fabarum* females after exposure to residues of the three insecticides on bean leaves weathered for various periods are provided in Table 1. There were significant differences among insecticides in all four post-treatment intervals (one day: *F* = 110.66, df = 2, *p* < 0.001; five days: *F* = 21.48, df = 2, *p* = 0.007; 16 days: *F* = 6.38, df = 2, *p* = 0.047; 30 days: *F* = 15.03, df = 2, *p* = 0.014). Notably, imidacloprid caused significantly higher mortality than the other two compounds even after 30 days post-treatment.

**Table 1.**
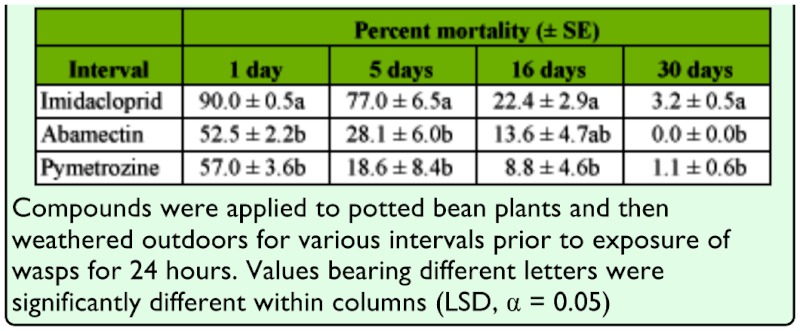
Residual toxicity of three pesticides to three day-old *Lysiphlebus fabarum* females following different post-treatment periods.

## Discussion

This study showed that both imidicloprid and abamectin were directly lethal to adult *L. fabarum*, especially 24 hours after application, with imidacloprid causing the highest mortality. These results are comparable to those of Vogt and Ternes (2005) who observed that residual contact with even very low rates of imidacloprid resulted in high mortality of *Aphelinus mali* (Haldeman) (Hymenoptera: Aphelinidae). Pymetrozine was the least toxic among the insecticides tested, a result consistent with the findings of Torres et al. (2003) who concluded that pymetrozine caused negligible mortality of *Aphelinus gossypii* Timberlake (Hymenoptera: Aphelinidae).

The residual toxicity of both abamectin and pymetrozine declined more rapidly with time than did that of imidicloprid. Similarly, Iqbal et al. (1996) found that the residual activity of abamectin against adult male *Diadegma semiclausum* Hellén (Hymenoptera: Ichneumonidae) declined rapidly when applied to Chinese cabbage at recommended field rates, with no detectable activity remaining two days after application. Likewise, Shipp et al. (2000) reported that the residual toxicity of avermectin b1 to *Aphidius colemani* Viereck (Hymenoptera: Braconidae) decreased significantly over time under greenhouse conditions.

Mummies of the parasitoid experienced appreciable mortality when exposed to all materials, but pymetrozine was the least toxic. Vogt and Ternes (2005) suggested that thiacloprid did not affect protected stages of *A. mali* within the woolly apple aphid, *Eriosoma lanigerum* (Hausmann) (Hemiptera: Aphididae), even when mummies were directly sprayed, likely due to protection afforded by the host integument. Therefore, when biological control fails to maintain aphids below threshold such that a pesticide application becomes necessary, a portion of the parasitoid population in the mummy stage may experience a functional refuge.

The use of pesticides may cause undesired effects on non-target beneficial organisms and lead to secondary pest outbreaks. To avoid this, the harmful effects of insecticides on natural enemies of the target pest should be minimized, either through careful timing of applications or the use of materials with selective activity. Based on IOBC criteria, imidacloprid would be categorized as slightly persistent, and both abamectin and pymetrozine as short-lived. We may infer that the latter insecticides are relatively compatible with biological control and that their more rapid loss of residual activity may also reduce the risk of exposure for consumers of agricultural goods. Since *L. fabarum* searches for hosts by walking over plant surfaces and seldom flies, this may increase its exposure to pesticide residues. However, if imidacloprid is applied as granules to the soil, or through chemigation (application in irrigation water), one can avoid direct parasitoid exposure to spray or residues on plant surfaces.

Pymetrozine has been shown to be compatible with the parasitoids *Aphelinus abdominalis* Dalman (Hymenoptera: Aphelinidae) and *Encarsia formosa* Gahan (Hymenoptera: Aphelinidae) when applied against aphids and whiteflies in greenhouses (Sechser et al. 1994). Moura et al. (2010) found that abamectin caused 10% mortality to adults of one population of the lacewing *Chrysoperla externa* (Hagen) (Neuroptera: Chrysopidae) and was harmless to those of another population. Abamectin is known to be labile to photo-oxidation in sunlight (Escalada et al. 2008) and pymetrozine was shown to have a half-life of only 3.5 days on broccoli plants (Shen et al. 2008). Thus, both abamectin and pymetrozine can be considered more IPM-compatible compounds than imidacloprid for broadcast application against *A. fabae* in legume crops.
